# Supplementary material for: How reliable are self-reported estimates of birth registration completeness? Comparison with vital statistics systems
Source: PLoS One. 2021 Jun 8;16(6):e0252140. doi: 10.1371/journal.pone.0252140 (PMC8186773; doi:10.1371/journal.pone.0252140)
Supplement: S1 Table — (DOCX) [file pone.0252140.s001.docx]

**S1 Table. CRVS completeness (calculated using UN birth estimates) and self-reported completeness (%), 12-23 months, by country**

| **Countries and areas** | **CRVS registration completeness** | **Self-reported completeness (%)** | | **CRVS data year** | **Self-reported data source, year** |
| --- | --- | --- | --- | --- | --- |
|  |  | **Certification**** | **Registration***** |  |  |
| Albania | 90 | 87 | 99 | 2013 | DHS 2017-18 |
| Argentina* | 100 | 99 | 100 | 2009 | MICS 2011-2012 |
| Armenia | 100 | 99 | 99 | 2014 | DHS 2015-2016 |
| Azerbaijan | 76 | 90 | 93 | 2003 | DHS 2006 |
| Barbados | 100 | 100 | 100 | 2007 | MICS 2012 |
| Bhutan | 95 | 100 | 100 | 2005 | MICS 2010 |
| Bosnia and Herzegovina | 100 | 98 | 100 | 2004 | MICS 2006 |
| Colombia | 90 | – | 98 | 2013 | DHS 2015 |
| Côte d'Ivoire | 59 | 59 | 71 | 2013 | MICS 2016 |
| Cuba | 100 | 100 | 100 | 2012 | MICS 2014 |
| Dominican Republic | 69 | 82 | 87 | 2012 | MICS 2014 |
| Egypt | 100 | 100 | 100 | 2012 | DHS 2014 |
| El Salvador | 100 | 87 | 99 | 2012 | ENS/MICS 2014 |
| Honduras | 96 | 95 | 97 | 2010 | DHS 2011-2012 |
| Jordan | 93 | 89 | 99 | 2015 | DHS 2017-2018 |
| Kazakhstan | 100 | 100 | 100 | 2013 | MICS 2015 |
| Kenya | 54 | 24 | 68 | 2012 | DHS 2014 |
| Kyrgyzstan | 94 | 98 | 99 | 2015 | MICS 2018 |
| Maldives | 91 | 93 | 100 | 2014 | DHS 2016-2017 |
| Mexico | 89 | 95 | 95 | 2012 | MICS 2015 |
| Mongolia | 100 | 100 | 100 | 2015 | MICS 2018 |
| North Macedonia | 100 | 99 | 100 | 2009 | MICS 2011 |
| Panama | 99 | 92 | 96 | 2011 | MICS 2013 KFR |
| Paraguay | 45 | 87 | 90 | 2013 | MICS 2016 |
| Philippines | 74 | 68 | 93 | 2015 | DHS 2017 |
| Saint Lucia | 99 | 72 | 95 | 2013 | MICS 2012 |
| Suriname | 100 | 94 | 99 | 2014 | MICS 2018 |
| Thailand* | 100 | 100 | 100 | 2011 | MICS 2015-2016 |
| Trinidad and Tobago | 89 | 92 | 100 | 2009 | MICS 2011 |
| Tunisia | 100 | 98 | 100 | 2011 | MICS 2018 |
| Ukraine | 100 | 100 | 100 | 2010 | MICS 2012 |
| Uruguay | 98 | 100 | 100 | 2012 | MICS 2013 |
| Uzbekistan | 94 | 100 | 100 | 2005 | MICS 2006 |

* For self-reported data, UNICEF state "Data differ from the standard definition or refer to only part of a country."(1)

**Reference**

1. UNICEF. Global Databases: Birth Registration. In: UNICEF, editor. Global Databases: Birth Registration. New York2020.
