# Supplementary material for: How reliable are self-reported estimates of birth registration completeness? Comparison with vital statistics systems
Source: PLoS One. 2021 Jun 8;16(6):e0252140. doi: 10.1371/journal.pone.0252140 (PMC8186773; doi:10.1371/journal.pone.0252140)
Supplement: S2 Table — (DOCX) [file pone.0252140.s002.docx]

**S2 Table. Absolute difference (self-reported completeness minus CRVS completeness) calculated using UN birth estimates (percentage points), 12-23 months, by country with unpublished data**

| **Countries** | **Absolute difference (Self-reported minus CRVS) (percentage points)** | |
| --- | --- | --- |
|  | **Self-reported certification** | **Self-reported registration** |
| Ghana | +4 | +18 |
| Malawi* | – | +5 |
| Myanmar | +5 | +9 |
| Rwanda | -20 | +32 |
| United Republic of Tanzania | +2 | +16 |
| Zambia | -5 | +5 |

Authors’ calculations. Country-years are Ghana: CRVS 2014, DHS 2014; Malawi: CRVS 2014, MICS 2013-14; Myanmar: CRVS 2013, DHS 2015-16; Rwanda: CRVS 2015, DHS 2014-15; United Republic of Tanzania: CRVS 2013, DHS 2015-16; Zambia: CRVS 2014, DHS 2018.
