# Supplementary material for: How reliable are self-reported estimates of birth registration completeness? Comparison with vital statistics systems
Source: PLoS One. 2021 Jun 8;16(6):e0252140. doi: 10.1371/journal.pone.0252140 (PMC8186773; doi:10.1371/journal.pone.0252140)
Supplement: S1 Text — (DOCX) [file pone.0252140.s003.docx]

**S1 Text. Country Publications**

Below is a list of country publications from which data MICS, DHS or CRVS data derived, in addition to that reported in the UNICEF database or birth registration database. For some of these countries, DHS or MICS data were extracted from online DHS or MICS microdata.(1, 2)

**MICS**

Argentina MICS 2011-12: Secretaría Nacional de Niñez, Adolescencia y Familia y Fondo de las Naciones Unidas para la Infancia. Argentina: Encuesta de Indicadores Múltiples por Conglomerados 2011/2012, Informe Final. Buenos Aires: Secretaría Nacional de Niñez, Adolescencia y Familia y Fondo de las Naciones Unidas para la Infancia, 2013.

Barbados MICS 2012: Barbados Statistical Service. Barbados Multiple Indicator Cluster Survey 2012: Final Report. Bridgetown, Barbados: BSS, 2014.

Bhutan MICS 2010: National Statistics Bureau. Bhutan Multiple Indicator Survey 2010. Thimphu, Bhutan: National Statistics Bureau, 2011.

Bosnia and Herzegovina MICS 2006: Directorate for Economic Planning of Bosnia and Herzegovina, the Ministry of Health and Social Protection of the Republika Srpska and the Ministry of Health of the Federation of Bosnia and Herzegovina. Multiple Indicator Cluster Survey 2006, Final Report. Sarajevo: Directorate for Economic Planning of Bosnia and Herzegovina, the Ministry of Health and Social Protection of the Republika Srpska and the Ministry of Health of the Federation of Bosnia and Herzegovina, 2007.

Côte d'Ivoire MICS 2016: Ministère de Plan, une Enquête Réalisée par L’Institut National de la Statistique et du Développement. La Situation des Femmes et des Enfants en Côte d'Ivoire Enquête à Indicateurs Multiples 2016 - MICS5. Abidjan: Ministère de Plan, une Enquête Réalisée par L’Institut National de la Statistique et du D éveloppement, 2017.

Cuba MICS 2014: Dirección de Registros Médicos y Estadísticas de Salud, Ministerio de Salud Pública. Encuesta de Indicadores Múltiples por Conglomerados. Cuba, 2014. Informe final. La Habana, Cuba: Dirección de Registros Médicos y Estadísticas de Salud, Ministerio de Salud Pública. Encuesta de Indicadores Múltiples por Conglomerados, 2014.

Dominican Republic MICS 2014: Oficina Nacional de Estadística y UNICEF. Encuesta Nacional de Hogares de Propósitos Múltiples - Encuesta de Indicadores Múltiples por Conglomerados 2014, Informe Final. Santo Domingo, República Dominicana: Oficina Nacional de Estadística, 2016.

El Salvador ENS/MICS 2014: Ministerio de Salud. Encuesta nacional de salud de Indicadores Múltiples por Conglomerados 2014. San Salvador: Ministerio de Salud, 2016.

Kazakhstan MICS 2015: The Statistics Committee of the Ministry of National Economy of the Republic of Kazakhstan (Statistics Committee of the MNE RK), the United Nations Children’s Fund (UNICEF), the United Nations Population Fund (UNFPA). 2015 Kazakhstan Multiple Indicator Cluster Survey, Final Report. Astana, Kazakhstan: The Statistics Committee of the MNE RK, UNICEF and UNFPA, 2016.

Kyrgyzstan MICS 2018: National Statistical Committee of the Kyrgyz Republic and UNICEF. Kyrgyzstan Multiple Indicator Cluster Survey 2018, Final Report. Bishkek, Kyrgyzstan: National Statistical Committee of the Kyrgyz Republic and UNICEF, 2019.

Mexico MICS 2015: Instituto Nacional de Salud Pública y UNICEF México. Encuesta Nacional de Niños, Niñas y Mujeres 2015 - Encuesta de Indicadores Múltiples por Conglomerados 2015, Informe Final. Ciudad de México, México: Instituto Nacional de Salud Pública y UNICEF México, 2016.

Mongolia MICS 2014: NSO. Social Indicator Sample Survey-2018, Survey Findings Report. Ulaanbataar, Mongolia: National Statistical Office of Mongolia, 2019.

Montenegro MICS 2018: Statistical Office of Montenegro (MONSTAT) and UNICEF. 2018 Montenegro Multiple Indicator Cluster Survey and 2018 Montenegro Roma Settlements Multiple Indicator Cluster Survey, Survey Findings Report. Podgorica, Montenegro: MONSTAT and UNICEF, 2019.

North Macedonia MICS 2011: Ministry of Health, Ministry of Education and Science, and Ministry of Labour and Social Policy of the Government of Republic of Macedonia. Republic of Macedonia Multiple Indicator Cluster Survey 2011, Skopje: Ministry of Health, 2012.

Panama MICS 2013 KFR. Contraloría General de la República. Encuesta de Indicadores Múltiples por Conglomerados de Panamá 2013, Resultados Principales Panamá, Panamá: Contraloría General, 2014.

Paraguay MICS 2016: Dirección General de Estadística, Encuestas y Censos y suministraron apoyo financiero y técnico; el Ministerio de Salud Pública y Bienestar Social y el Fondo de las Naciones Unidas para la Infancia (UNICEF). La Encuesta de Indicadores Múltiples por Conglomerados, MICS Paraguay, fue realizada en el 2016. Asunción: Dirección General de Estadística, Encuestas y Censos, 2017.

Republic of Moldova MICS 2012: National Centre of Public Health of the Ministry of Health of the Republic of Moldova, United Nations Children’s Fund (UNICEF). 2012 Republic of Moldova Multiple Indicator Cluster Survey, Final Report. Chișinău, Republic of Moldova: National Centre of Public Health, 2014.

Saint Lucia MICS 2012: Ministry of Social Transformation, Local Government and Community Empowerment and Central Statistics Office, Saint Lucia Multiple Indicator Cluster Survey 2012: Final Report, Castries, Saint Lucia: Ministry of Social Transformation, Local Government and Community Empowerment, 2014.

Suriname MICS 2018: Ministry of Social Affairs and Public Housing. Suriname Multiple Indicator Cluster Survey 2018, Survey Findings Report. Paramaribo, Suriname: Ministry of Social Affairs and Public Housing, 2019.

Thailand MICS 2015-2016. National Statistical Office and United Nations Children’s Fund. Thailand Multiple Indicator Cluster Survey 2015-2016, Final Report. Bangkok: NSO and UNICEF, 2016.

Trinidad and Tobago MICS 2011. Ministry of Social Development and Family Services, Central Statistical Office and UNICEF. Trinidad and Tobago Multiple Indicator Cluster Survey 2011, Key Findings & Tables. Port of Spain, Trinidad and Tobago: Ministry of Social Development and Family Services, Central Statistical Office and UNICEF, 2012.

Tunisia MICS 2018: Institut National de la Statistique et UNICEF. 2019 Enquête par grappes à indicateurs multiples (MICS), 2018, Rapport Final. Tunisie: le Ministère du Développementde l’Investissement et de la Coopération Internationale (MDICI), 2020.

Ukraine MICS 2012: State Statistics Service and Ukrainian Center for Social Reforms. Ukraine Multiple Indicator Cluster Survey 2012, Final Report. Kyiv, Ukraine: State Statistics Committee and the Ukrainian Center for Social Reforms, 2013.

Uruguay MICS 2013. UNICEF y MIDES. Uruguay. Encuesta de Indicadores Múltiples por Conglomerados 2013, Informe final. Montevideo: UNICEF y MIDES, 2015.

Uzbekistan MICS 2006. UNICEF and State Statistical Committee of the Republic of Uzbekistan. Uzbekistan Multiple Indicator Cluster Survey 2006, Final Report. Tashkent, Uzbekistan: UNICEF, 2007.

**DHS**

Albania DHS 2017-18: Institute of Statistics, Institute of Public Health, and ICF. Albania Demographic and Health Survey 2017-18. Tirana, Albania: Institute of Statistics, Institute of Public Health, and ICF, 2018.

Armenia DHS 2015-2016: National Statistical Service [Armenia], Ministry of Health [Armenia], and ICF. Armenia Demographic and Health Survey 2015-16. Rockville, Maryland, USA: National Statistical Service, Ministry of Health, and ICF, 2017.

Azerbaijan DHS 2006: State Statistical Committee (SSC) [Azerbaijan] and Macro International Inc. Azerbaijan Demographic and Health Survey 2006. Calverton, Maryland, USA: State Statistical Committee and Macro International Inc, 2008.

Colombia DHS 2015: Minsalud, Profamila and Todos por un Nuevo Pais. Encuesta Nacional de Demografía y Salud. Bogota: Minsalud, 2016.

Egypt DHS 2014 :Ministry of Health and Population [Egypt], El-Zanaty and Associates [Egypt], and ICF International. Egypt Demographic and Health Survey 2014. Cairo, Egypt and Rockville, Maryland, USA: Ministry of Health and Population and ICF International, 2015.

Ghana DHS 2014: Ghana Statistical Service (GSS), Ghana Health Service (GHS), and ICF International. Ghana Demographic and Health Survey 2014. Rockville, Maryland, USA: GSS, GHS, and ICF International, 2015.

Honduras DHS 2011-2012. Secretaría de Salud [Honduras], Instituto Nacional de Estadística (INE) e ICF International. Encuesta Nacional de Salud y Demografía 2011-2012. Tegucigalpa, Honduras: SS, INE e ICF International, 2013.

Jordan DHS 2017-2018. Department of Statistics (DOS) and ICF. Jordan Population and Family and Health Survey 2017-18. Amman, Jordan, and Rockville, Maryland, USA: DOS and ICF, 2019.

Kenya DHS 2014. Kenya National Bureau of Statistics. Kenya Demographic and Health Survey 2014. Nairobi, Kenya: Kenya National Bureau of Statistics, 2015.

Maldives DHS 2016-2017. Ministry of Health (MOH) [Maldives] and ICF. Maldives Demographic and Health Survey 2016-17. Malé, Maldives, and Rockville, Maryland, USA: MOH and ICF, 2018.

Myanmar DHS 2015-2016. Ministry of Health and Sports (MoHS) and ICF. Myanmar Demographic and Health Survey 2015-16. Nay Pyi Taw, Myanmar, and Rockville, Maryland USA: Ministry of Health and Sports and ICF, 2017.

Philippines DHS 2017. Philippine Statistics Authority (PSA) and ICF. Philippines National Demographic and Health Survey 2017. Quezon City, Philippines, and Rockville, Maryland, USA: PSA and ICF, 2018.

Rwanda DHS 2014-2015. National Institute of Statistics of Rwanda (NISR) [Rwanda], Ministry of Health (MOH) [Rwanda], and ICF International. Rwanda Demographic and Health Survey 2014-15. Rockville, Maryland, USA: NISR, MOH, and ICF International, 2015.

United Republic of Tanzania DHS 2015-2016. Ministry of Health, Community Development, Gender, Elderly and Children (MoHCDGEC) [Tanzania Mainland], Ministry of Health (MoH) [Zanzibar], National Bureau of Statistics (NBS), Office of the Chief Government Statistician (OCGS), and ICF. Tanzania Demographic and Health Survey and Malaria Indicator Survey (TDHS-MIS) 2015-16. Dar es Salaam, Tanzania, and Rockville, Maryland, USA: MoHCDGEC, MoH, NBS, OCGS, and ICF, 2016.

Zambia DHS 2018. Zambia Statistics Agency, Ministry of Health (MOH) Zambia, and ICF. Zambia Demographic and Health Survey 2018. Lusaka, Zambia, and Rockville, Maryland, USA: Zambia Statistics Agency, Ministry of Health, and ICF, 2019.

**CRVS**

Bolivia: Instituto Nacional de Estadistica. Población y Hechos Vitales. La Paz, Bolivia: INE, 2020. <https://www.ine.gob.bo/index.php/registros-administrativos-sociales/>

Cote d’Ivoire. Ministere de l’Interieur et de la Securite. Anuair Statistique d’etat Civil 2017. Abidjan: Ministere de l’Interieur et de la Securite, 2018.

Dominican Republic: Oficina Nacional de Estadística. Nacimientos. Santo Domingo, Dominican Republic: ONE, 2020. <https://www.one.gob.do/demograficas/estadisticas-vitales/nacimientos>

Honduras: Instituto Nacional de Estadistica. El Sistema de Estadísticas Vitales en Honduras 2010-12. Honduras: INE, 2013.

Kenya: Kenya National Bureau of Statistics. Statistical Abstract 2014. Nairobi: Kenya National Bureau of Statistics, 2015.

Nicaragua: Instituto Nacional de Información de Desarrollo. Compendio Estadísticas Vitales 2007-2010. Managua, Nicaragua: INIDE, 2011.

Paraguay: Dirección General de Estadística, Encuestas y Censos. Estadísticas Vitales del Paraguay 2018. Ascuncion: DGEEC, 2019.

Peru: Instituto Nacional de Estadística e Informática (INEI). Perú: Natalidad, Mortalidad y Nupcialidad, 2014. Lima, Peru: INEI, 2015.

Thailand: Thai Health Information Standards Development Center (THIS) Health Systems Research Institute (HSRI)Review of National Civil Registration and Vital Statistics Systems: A case study of Thailand. Nonthaburi, Thailand, THIS HSRI, 2013.

**References**

1. ICF. The Demographic and Health Surveys (DHS) Program Rockville, MD2020 [Available from: <https://dhsprogram.com/>.

2. UNICEF. UNICEF MICS Surveys New York2020 [Available from: <https://mics.unicef.org/surveys>.
